# 

**Published:** 1890-03-29

**Authors:** 


					ottered at the General Post Office as a Newspaper for
transmission in the United Kingdom and Abroad,
Per Annum, 6s, 63.; post free.
Monthly Parts, 6d.; post free, 8d.
Ditto per Annum, 8s, J post free.

				

